# Daily outdoor PM_2·5_ exposure characterization and road edge effect during a 2022 – 2023 monitoring campaign in Gambia, Kenya and Mozambique

**DOI:** 10.1016/j.aeaoa.2026.100463

**Published:** 2026-05-22

**Authors:** Terence Darlington Mushore, Handsome Bongani Nyoni, Laura Munthali, Sibusisiwe Audrey Makhanya, Laurine Chikoko, Anotida Marrian Chapunza, Stanley Luchters, Matthew Chersich, Fortunate Machingura, Liberty Makacha, Benjamin Barratt, Hiten D. Mistry, Marie Laure Volvert, Cherlynn Dumbura, Umberto D’Alessandro, Anna Roca, Esperana Sevene, Herminio Cossa, Marleen Temmerman, Angela Koech, Peter von Dadelszen, Tamara Govindasamy, Prestige Tatenda Makanga, The PRECISE Network, The HEAT Centre

**Affiliations:** a Place Alert Labs, Surveying and Geomatics Department, Faculty of the Built Environment Midlands State University, Gweru, Zimbabwe; b Climate Environment and Health Department, Center for Sexual Health and HIV AIDS Research (CeSHHAR), Harare, Zimbabwe; c IBM Research Africa, South Africa; d Department of International Public Health, Liverpool School of Tropical Medicine (LSTM), UK; e Department of Women and Children’s Health, School of Life Course and Population Sciences, Faculty of Life Sciences and Medicine, King’s College London, UK; f Environmental Research Group, MRC Centre for Environment and Health, Michael Uren Biomedical Engineering Hub, White City Campus, Imperial College London, University of Zimbabwe, UK; g Department of Obstetrics and Gynaecology, University of British Columbia, Vancouver, British Columbia, Canada; h ICRH, Department of Public Health and Primary Care, Ghent University, Belgium; i Wits Planetary Health Research, University of the Witwatersrand, Johannesburg, 2193, South Africa; j Department of Space Science and Applied Physics, Faculty of Science, University of Zimbabwe, Zimbabwe; k School of Agriculture and Environmental Sciences, Department of Climate Change and Education, University of the Gambia, Republic of the Gambia; l Barcelona Institute of Global Health, Barcelona, Spain; m Manhiça Health Research Center, Mozambique; n Centre of Excellence in Women and Child Health, Aga Khan University, Nairobi, Kenya

**Keywords:** Emission, Pollution, Air quality, Edge effect, Health outcomes, Particulate matter

## Abstract

Air pollution is a major contributor to mortality and adverse health outcomes, particularly in pregnant women and children, with vehicular emissions as a key source. This study aimed to characterize long-term exposure to fine particulate matter (PM_2·5_), evaluate potential health risks, and quantify the contribution of road transport and proximity to roads in The Gambia, Kenya, and Mozambique. Multi-temporal personal exposure data were interpolated into continuous spatial surfaces and aggregated into daily averages, which were then compared with World Health Organization (WHO) guidelines to assess potential health impacts. Multi-distance buffers at 10 m intervals up to 1000m from major roads were created to examine pollutant variation with road proximity. Overlay analyses and linear regression models were applied to quantify the road-edge effect on pollution exposure. The Inverse Distance Weighting interpolation technique produced accurate PM_2·5_ field in all sites (R2>0.55, MAE<4 μg/m3 and RMSE<10 μg/m3) based on leave-one-out cross-validation. PM_2·5_ concentrations were highest in The Gambia across all land use and land cover (LULC) types, reflecting localized industrial and vehicular emissions. Elevated pollutant levels were observed in herbaceous wetlands, bare land, and built-up areas. Most sites across all three countries exceeded WHO recommended thresholds, indicating significant health risks. PM_2·5_ decreased with distance from roads in Rabai (Kenya) and Mozambique, while in other sites it increased away from roads. These findings highlight the combined influence of multiple local sources, including traffic, fire smoke, wetlands, and industry, on pollutant exposure. The study demonstrates the utility of WHO thresholds for health risk assessment and shows how road proximity affects pollutant dispersal. The results provide critical insights for modeling air pollution exposure and informing mitigation strategies in sub-Saharan Africa.

## 1. Introduction

Air pollution is among the leading causes of adverse health outcomes globally. Contributing factors to elevated concentrations of pollutants include anthropogenic activities such as landscape fires, heating and cooking as well as emissions associated with increased density of vehicles along major road, agricultural and industrial activities (Kodama et al., 2002; Malagón-Rojas et al., 2022). Exposure to pollutants such as course and superfine particulate matter (PM_10_ and PM_2·5_) can cause respiratory and lung diseases such as lung cancer and asthma, obstructive pulmonary diseases, immune system problems and other health challenges (Kodama et al., 2002; Sheng et al., 2019). The same exposure has also been associated with vasoconstriction and resultant hypertension or decreased blood flow (Kobza and Geremek, 2017). In Bogota, about 21,000 deaths between 2012 and 2022 were attributable to air pollution (Malagón-Rojas et al., 2022). Studies have also associated exposure to air pollution with adverse maternal, newborn and children health such as pre-term birth, hypertension, low birth weight and cardio-vascular diseases (Aung et al., 2019; Bekkar et al., 2020; Bell et al., 2008; Ekland et al., 2021; Jiang et al., 2023). Besides human health, air quality also affects buildings, biodiversity, historical monuments, plants and visibility (Munir and Habeebullah, 2018). Understanding air quality patterns is an important step towards efforts to reduce emissions and alleviate adverse health effects.

The World Health Organization (WHO) provides and regularly updates air quality guidelines to assist governments in policy formulation and actions to reduce pollutant concentrations in the atmosphere. The guidelines are derived through rigorous systematic review of evidence and meta-analyses of quantitative effects taking into account latest body of knowledge on health impacts of different pollutants (World Health Organization, 2021). The guidelines consist of recommendations of concentration levels for both short- and long-term exposure for a number of key pollutants (World Health Organization, 2021). They also present interim targets which guide reduction efforts towards the ultimate and timely achievement of Air Quality Guidelines (AQG) for regions where lowest recommended levels are exceeded. World Health Organization (2021) asserts that concentrations below AQG are desirable as they coincide with minimal or no air quality induced adverse health outcomes. In view of this, stepwise efforts should be made to reduce pollution concentrations towards an interim target until levels are brought to and maintained below AQG. Li et al. (2020) stressed that additional efforts were needed to bring atmospheric pollutant concentrations down to acceptable levels globally. Studies should take advantage of geospatial techniques to monitor short- and long-term pollution exposure and assess the extent to which World Health Organizations recommendations for different key pollutants are achieved.

Road transport is among main sources of atmospheric pollutants such as, municipal and industrial sources related to heating and disposal of waste (Szopińska et al., 2022). Primary pollutants emitted by motor vehicles are CO, NO_2_ and particulate matter (Kobza and Geremek, 2017). Particles with diameter ranging between 40 and 60 nm and between 20 and 130 nm are emitted by gasoline and diesel engines, respectively (Lin et al., 2016). An increase in the number of vehicles leads to decrease in speed of vehicle and increased time of releasing pollutants into the atmosphere (Sheng et al., 2019). In Japan in 2002, more than 30% of roadside monitors recorded Particulate Matter values which were higher than recommended standards (Kodama et al., 2002). In Nanjing in China, vehicular emissions contributed to 31% of all emissions (Sheng et al., 2019). Vehicles directly pollute the air due to combustion of liquid fuel and wearing of brakes and tires (Szopińska et al., 2022). Indirect mechanisms include condensation of gaseous substances emitted in exhaust fumes as well as secondary lifting of particles deposited on the road surface (Huertas Cardozo and Prato Sánchez, 2019; Szopińska et al., 2022). Course and ultra-fine particles exist in both paved and unpaved roads while even in the absence of combustion, traffic also contribute to these particulates through non-exhaust emissions (Huertas Cardozo and Prato Sánchez, 2019). Studies showed that pollutant levels during Covid-19 lockdown decreased due to reduced contribution from direct vehicular emissions (Li et al., 2020; Nakada and Urban, 2020). In Brazil, vehicular emissions (NO, NO_2_ and CO) reduced by between 54 and 77% in urban areas during the Covid-19 lockdown (Nakada and Urban, 2020). The effect of vehicular emission is thus remarkable requiring constant assessment alongside implications on WHO recommended emission reduction guidelines.

Studies have shown strong links between pollutant concentration (course or fine) and distance from main roads with a general decrease with distance from the road (Kobza and Geremek, 2017; Kodama et al., 2002; Lawson et al., 2011; Munir and Habeebullah, 2018). (Cardozo and Sánchez (2019)) stressed that people who settle within 100 to 500m from main roads are exposed to significant vehicular emissions with marked reductions beyond this point. They observed that NO_2_ concentration showed an exponential decrease function at the edge of the road, an s-shape downwind and a flat shape away (0.5 km) from the roads. In North Carolina in USA, Lin et al. (2016) observed that people living within 100 to 300m of high traffic roadway experienced higher ultra-fine particle exposure compared to background level. In Japan cities in 2002, NO_2_ concentration decreased with distance from roadside (Kodama et al., 2002). Short term and long-term patterns differ as short term exposures are mainly influenced by traffic patterns while long term by weather parameters (Munir and Habeebullah, 2018). Magnitude and extent of pollution from vehicles depend on type of power of the engine, operating condition of the engine, applied measures to reduce pollution, meteorological conditions, composition of fuel as well as type and condition of the road surface among others (Szopińska et al., 2022). Other factors such as roadside vegetation, forests and industrial activities also affect deposition and aerodynamic transfer of pollutants from roads to surrounding areas (Abhijith and Gokhale, 2015; Viippola et al., 2018; Vranckx et al., 2015). Although most evidence comes from North America, Europe, and Asia, similar dynamics are expected in African cities where road traffic is increasing rapidly, and monitoring capacity remains limited.

Due to scarcity of observations, analysis of air quality and potential impacts has remained minimal in Africa compared to other continents. There is need to assess the extent to which pollutant levels deviate from recommended concentrations for minimal adverse health effects. The road edge effect on air quality also needs to be understood in different areas especially the African context. Such an understanding will help Africa to develop sustainably and reduce the burden of environmental challenges given that the continent still suffers a myriad of problems with limited coping capacity. Area specific analysis of the effect of proximity to roads on exposure is critical for designing solutions to tackle adverse results of exposure to road transport related emissions. Analysis is also important for the comparison of road edge pollution with concentrations away from the road. Although previous studies mostly assessed air quality and effect of main roads in urban areas, the rural context and experiences in the rural-urban continuum are also important, especially in Africa where most of the population is rural while there is rapid urban expansion. In view of the above, the study (i) seeks assess daily PM_2·5_ levels in three study sites in the Gambia, Kenya and Mozambique against World Health Organization targets and recommendations and (ii) determine the effect of proximity to main roads on exposure to PM_2·5_ using geospatial analysis techniques. The findings contribute to assessing exposure trends, identifying pollution hotspots, and informing policy interventions aimed at reducing air pollution exposure and its associated health risks.

## 2. Datasets, methods and analysis

### 2.1. Study area

**Fig. 1.**
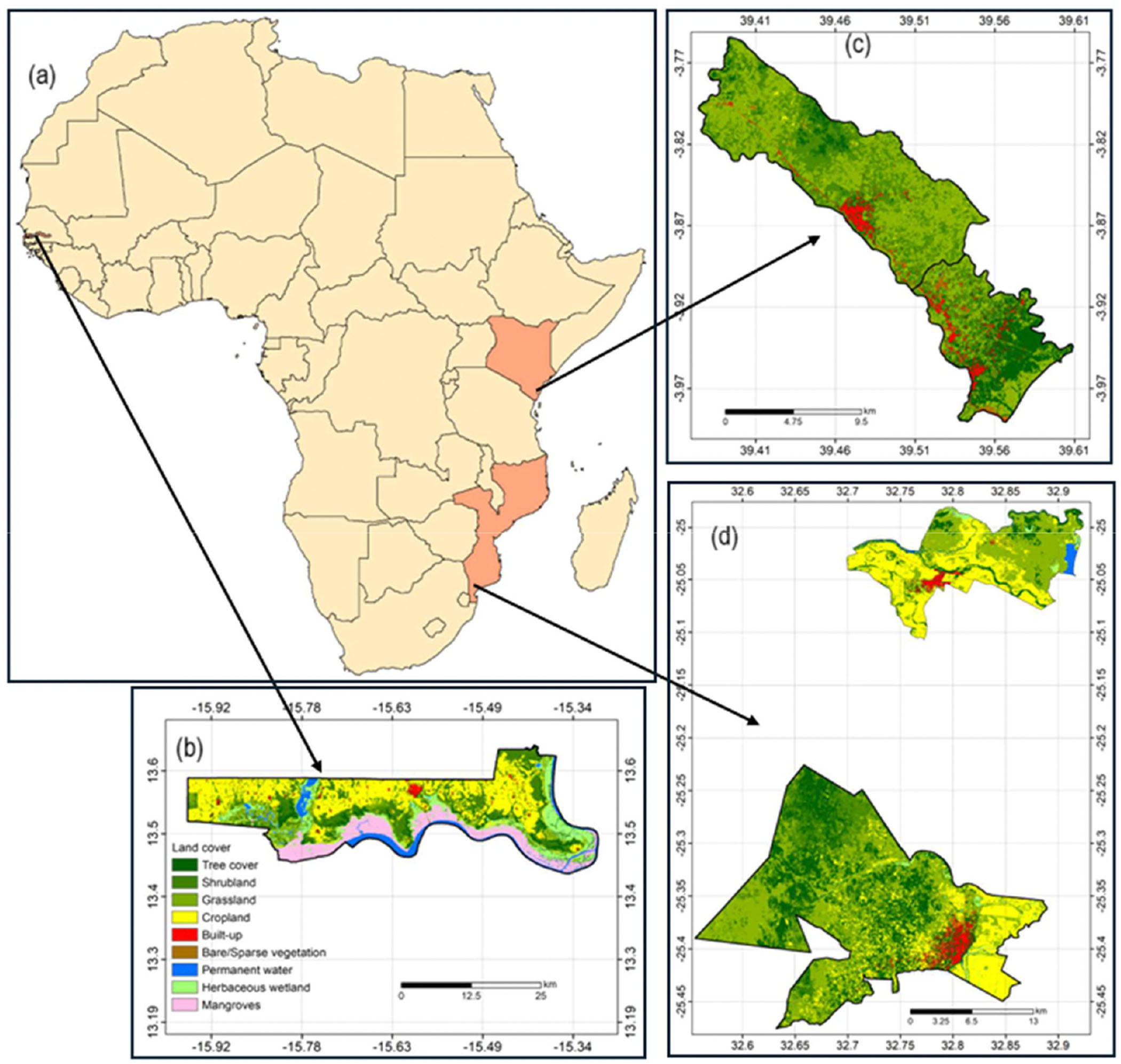
Study areas and landcover characteristics in The Gambia, Kenya, and Mozambique. Panel (a) shows the location of the study sites within Africa. Panel (b) presents the landcover map of the North Bank Region in The Gambia, panel (c) shows Mariakani and Rabai in Kenya, while panel (d) shows Manhiça and Xinavane in Mozambique. Landcover classes were derived from the 10 m European Space Agency (ESA) WorldCover 2021 dataset. Coordinates are presented in geographic latitude and longitude. Maps were generated in ArcGIS Pro.

The study was conducted in The Gambia, Kenya, and Mozambique (Fig. 1a). Landcover data were derived from the 10m European Space Agency (ESA) Global Landcover Map (Zanaga et al., 2022; https://worldcover2021.esa.int/download), released in 2021 with 75% global accuracy suitable for regional analysis. In The Gambia, the North Bank Region (13°29′10″N, 15°5′48″W) is characterized by erratic rainfall, poor soils, and low fertility, with a wet season from June to October and a dry season from November to March. Average temperatures are 30 °C (dry) and 27 °C (wet) (Sonko et al., 2016). Kerewan, the regional capital, receives the least rainfall (Kutir et al., 2015). Croplands and mangroves dominate, with water bodies along the southern border and built-up areas in central and eastern zones. In Kenya, two wards in Kilifi County, Mariakani and Rabai, were studied (Khaemba et al., 2023). Mariakani (3°52′ S, 39°28′ E) is a marine industrial area (Nthiga, 2016; Onyatta et al., 2016) where grasslands cover over 50% and shrubs and trees about 25% (Fig. 1c). Rainfall patterns follow the Indian monsoons with two rainy seasons (Camberlin and Planchon, 1997). Rabai, about 20 km northwest of Mombasa, has undulating terrain (110–125 m) and a tropical savanna climate, with 120,813 residents in 2019. Agriculture is mostly subsistence, producing cassava, maize, vegetables, coconuts, cashews, and pineapples. It also hosts a thermal power plant and has extensive road links. Croplands, grasslands, and trees dominate the area. In Mozambique, the site was Manhiça District (373 km^2^) in Maputo Province, about 80 km north of Maputo, with a population of ≈266,435 (Lima et al., 2025; Sacoor et al., 2013; Acácio et al., 2018). The flat terrain supports sugarcane, banana, and rice farming, with two sugar estates, Maragra and Xinavane (Curto et al., 2024; Munguambe et al., 2011). Rainfall averages 900–1100 mm (Sacoor et al., 2013). ESA maps show trees and grasslands in the west and croplands, wetlands, and built-up zones in the east.

### 2.2. Data processing and quality control

The study used personal exposure data from the PREgnancy Care Integrating Translational Science, Everywhere (PRECISE) network project in The Gambia, Kenya, and Mozambique (Craik et al., 2024). Data were collected from 343 pregnant women carrying personal exposure monitors at 1-min intervals in the three countries. Data were collected from 31 March 2022 to 31 January 2023 in The Gambia, 9 March to 13 December 2022 in Kenya, and 4 May to 11 October 2022 in Mozambique. Raw personal exposure data were recorded at 1-min intervals and synchronized with GPS location data. While PRECISE captured both indoor and outdoor exposure, this study focused on outdoor exposure and mobility. Data cleaning followed standardized procedures to ensure reliability of exposure estimates. Steps applied involved (i) removal of implausible pollutant values exceeding instrument operational limits (ii) exclusion of records with missing or invalid GPS coordinates (iii) filtering of duplicate timestamps and corrupted entries (iv) participant-day inclusion criteria requiring at least 80% valid recording time to ensure representativeness and (v) temporal alignment of pollutant and GPS datasets prior to outdoor classification. Outdoor exposure periods were identified using a movement speed threshold (>1.5 m/s) derived from consecutive GPS coordinates. This classification represents mobility-related or out-of-building exposure, including walking and transit periods. This threshold is intentionally conservative and exceeds the static-cluster threshold (~0.83 m/s) used in GPS-based time–activity classification studies (Wu et al., 2011), thereby minimizing misclassification of indoor or stationary periods as outdoor. Reported human walking speeds typically range from 1.0 to 1.8 m/s (Byrne et al., 2011; Loh et al., 2019), supporting the use of 1.5 m/s to identify sustained outdoor movement. While this approach reduces contamination from indoor measurements, it may not perfectly distinguish all microenvironments. To improve the robustness of this classification, a spatial validation step was implemented using footprint building polygons from OpenStreetMap. Building geometries were retrieved through the Overpass API and used to perform point-in-polygon analysis to determine whether monitoring points occurred inside or outside building footprints. Speed-based classifications were compared with building-based labels using confusion matrix analysis, and discrepancies were corrected using the building-derived classification as ground truth to minimize misclassification of indoor and outdoor environments. After applying these filtering and validation procedures, the recorded observations were retained for subsequent exposure analysis.

### 2.3. Annual PM_2.5_ exposure mapping

High-resolution outdoor location-time data were interpolated into spatial-temporal pollutant layers and aggregated as daily averages. Prior to interpolation, several preprocessing procedures were applied to ensure data quality and computational feasibility. Spatial outliers were removed using a percentile-based filtering approach that excluded observations within the lower and upper 2.5% tails of the longitude and latitude distributions. This ensured that only measurements within the main study domain were retained. To maintain computational feasibility while preserving temporal representativeness, data were temporally sampled to hour using dwell time, representing peak outdoor activity periods when exposure is most relevant. Inverse Distance Weighting (IDW) was applied to generate continuous surface maps of PM_2·5_ from daily outdoor measurements. Exposure points were imported as a consistent projected coordinate system, then IDW was applied using the Spatial Analyst toolset. Inverse Distance Weighting (IDW) interpolation was selected for its simplicity, computational efficiency, and ability to preserve measured values at sampling locations. The method assumes that observations closer to a prediction location exert stronger influence than those farther away. Predicted concentrations were calculated using a distance-weighted formulation (Equation (1) and Equation (2)):

[1] $$Z\left(x_{0}\right)=\frac{\sum w_{i}Z\left(x_{i}\right)}{\sum w_{i}},$$

where weights $w_{i}=1/d{\left(x_{0},x_{i}\right)}^{p}$ [2] are derived from the euclidean distance between observation and prediction locations. Concentrations were set as Z-values, with a defined search radius and neighboring points. A power parameter of 2 was applied to balance local influence with spatial smoothing, and raster resolution was optimized to maintain spatial detail while avoiding excessive computational load.

#### 2.3.1. Spatial interpolation validation and uncertainty assessment

To evaluate the robustness of the Inverse Distance Weighting (IDW) interpolation surfaces, leave-one-out cross-validation (LOOCV) was conducted separately for PM_2·5_ in each study site. In this approach, each observation was sequentially removed and its value predicted using the remaining dataset through the IDW model with a power parameter of 2, generating predicted–observed pairs for validation. A fixed search radius was applied with a minimum of 8 and maximum of 15 neighboring points to ensure stability of predictions while preserving local variability. To account for temporal variability in air pollutant concentrations, cross-validation was performed independently for each hourly period rather than pooling all observations across time, and only time periods with at least 30 valid measurements were included to ensure stable prediction estimates. Interpolation accuracy was assessed using four performance metrics: coefficient of determination (R^2^), Root Mean Square Error (RMSE), Mean Absolute Error (MAE), and Mean Error (ME), which collectively evaluate model fit, average prediction magnitude, typical error, and systematic bias. Validation statistics were aggregated across time periods for each country, pollutant combination and observed versus predicted scatter plots with 1:1 reference lines were produced to visually assess prediction performance.

### 2.4. Potential health impact assessment

#### 2.4.1. Comparison of daily pollutant levels with reduction targets

The WHO has established short- and long-term targets to reduce PM_2·5_ exposure, with achievement of these targets linked to reductions in adverse health outcomes (World Health Organization, 2021). WHO air quality guidelines provide quick and convenient criteria for assessing and estimating potential and broad scale health burdens associated with air quality hence the use in this study. They enable assessment of potential implications of air quality on human health even without digging into health data in the presence of air quality data. In this study, we focused on the daily targets for both pollutants. Table 1 summarizes the targets, showing the pollutant levels below which mean daily concentrations are aimed at falling in efforts to lower associated health risks. Using daily average pollutant maps, we evaluated and spatially mapped whether each pixel met or exceeded the threshold for each target, allowing assessment of compliance and potential public health benefits at a fine spatial scale.

#### 2.4.2. Mapping extent to which target to eliminate or significantly reduce adverse health outcomes from exposure to pollutants is satisfied

The WHO predicts that adverse health outcomes will be eliminated or extremely reduced if individual daily average PM_2·5_ levels are reduced to values below 15 μg/m^3^. We assessed the extent to which daily pollutant levels in the three countries are compared with this target. Places with values below the thresholds were classified as meeting the requirements for each pollutant. This was done per landcover in each study site in Gambia, Kenya and Mozambique. The analysis provided a measure of the extent to which each pollutant contributed to the adverse health effects in the area.

### 2.5. Determination of road edge effect on PM_2.5_ concentrations

Interpolated PM_2·5_ raster surfaces were integrated with road network data to quantify spatial gradients in pollutant concentrations relative to road proximity. Concentric multi-ring buffers were generated at 10m intervals from road edges up to 1000m, consistent with the 10m spatial resolution of the ESA land cover dataset. The 1000 m threshold was selected to capture near-road dispersion effects while minimizing interference from non-traffic emission sources. For each buffer distance, mean pollutant concentrations were extracted using zonal statistics in ArcGIS Pro. Ordinary least squares (OLS) linear regression models (Equation (3)) were then fitted for each site and pollutant.

[3] $$C=\beta_{0}+\beta_{1}D$$

where C is the mean buffer concentration (μg m^−3^), *β*_1_ is the slope and D is distance from the road (m). To enhance interpretability, regression slopes are reported per 100m increase in distance. Model outputs included slope (per 100m), 95% confidence intervals (CI), coefficient of determination (R^2^), Pearson correlation coefficient (r), and p-values. Negative slopes indicate decreasing concentrations with increasing distance (classical road-edge dispersion), whereas positive slopes indicate increasing concentrations away from roads.

## 3. Results

### 3.1. Segmentation of outdoor and indoor PM2.5 exposure in the Gambia, Kenya and Mozambique study sites

**Fig. 2.**
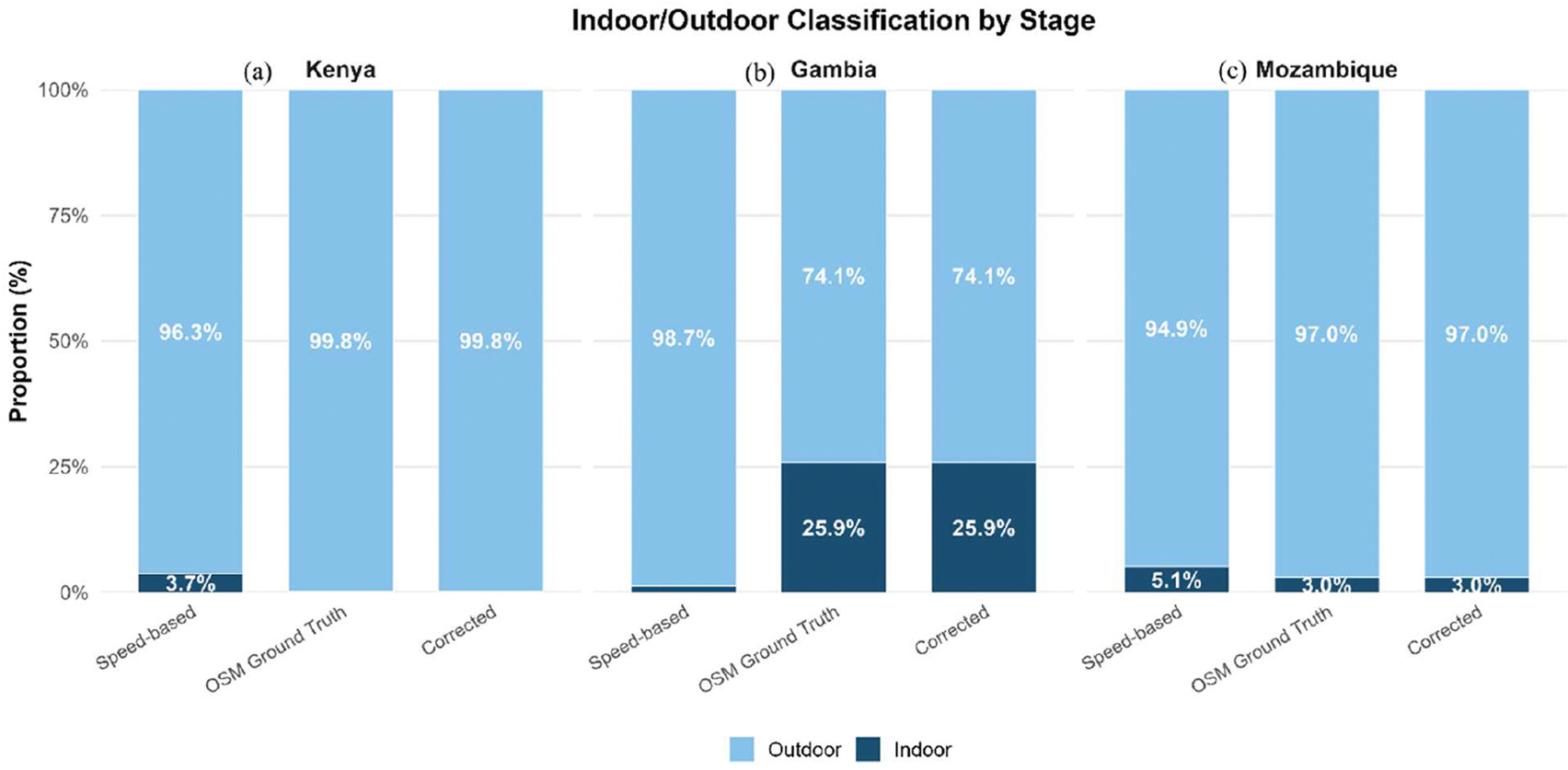
Indoor and outdoor PM_2·5_ exposure classification and validation results for The Gambia, Kenya, and Mozambique based on GPS movement speed and building-footprint spatial validation. Outdoor exposure periods were initially identified using a movement threshold greater than 1.5 m s^−1^. OpenStreetMap building polygons were subsequently used to validate indoor and outdoor classifications through point-in-polygon analysis. PM_2·5_ = particulate matter with aerodynamic diameter ≤2.5 μm.

Fig. 2 summarizes the results of the two-stage segmentation and validation analysis used to classify indoor and outdoor exposure observations across The Gambia (a), Kenya (b), and Mozambique (c). A total of 1,728,269 observations were analyzed in The Gambia, 1,061,833 in Kenya, and 571,055 in Mozambique. The initial speed-based classification indicated that most exposure measurements occurred outdoors, identifying 1,705,830 outdoor observations in The Gambia (98.7%), 1,022,582 in Kenya (96.3%), and 541,739 in Mozambique (94.9%). However, spatial validation using building footprints from OpenStreetMap revealed notable differences in the true indoor–outdoor distribution. In Kenya, only 1654 observations were located within buildings while 1,060,179 occurred outside, indicating that nearly all recorded exposures occurred outdoors. In The Gambia, a substantially larger share of observations occurred within buildings, with 446,865 indoor points and 1,281,404 outdoor points, corresponding to approximately 25.9% indoor and 74.1% outdoor exposure. Mozambique showed an intermediate pattern with 17,289 indoor observations and 553,766 outdoor observations, representing approximately 3% indoor and 97% outdoor exposure. Comparison between speed-based classifications and building-based validation revealed varying levels of misclassification, with Kenya showing relatively low error (40,803 misclassified observations; 4%), Mozambique showing moderate error (46,239 observations; 8%), and The Gambia showing substantially higher misclassification (459,392 observations; 27%), primarily due to false outdoor classifications where indoor movements were incorrectly labeled as outdoor. After correcting discrepancies using the building-based classification as ground truth, the final exposure distributions remained strongly dominated by outdoor environments in Kenya (≈100%) and Mozambique (≈97%), while The Gambia retained a notable indoor component (≈26%), indicating important differences in exposure environments across the study sites.

### 3.2. Daily interpolation performance cross validation in the Gambia, Kenya, and Mozambique study sites

**Fig. 3.**
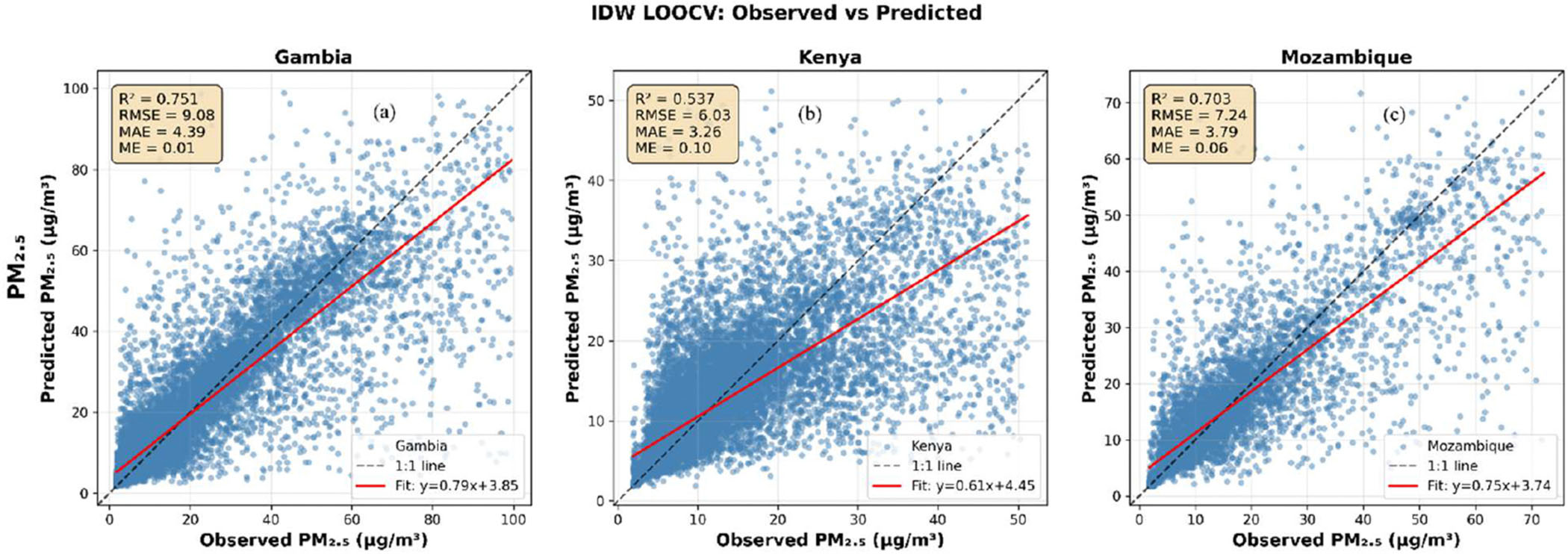
Leave-one-out cross-validation (LOOCV) performance of Inverse Distance Weighting (IDW) interpolation for PM_2·5_ in The Gambia, Kenya, and Mozambique showing observed versus predicted concentrations. The dashed 1:1 line represents perfect agreement between observed and predicted values. R^2^ = coefficient of determination; RMSE = Root Mean Square Error; MAE = Mean Absolute Error; ME = Mean Error. Concentrations are expressed in μg m^−3^.

Fig. 3 shows the leave-one-out cross-validation (LOOCV) performance of the IDW interpolation for PM_2·5_ across the three study sites (The Gambia, Kenya, and Mozambique), with observed versus predicted concentrations plotted alongside the 1:1 reference line. For PM_2·5_, the interpolation demonstrated strong predictive performance in The Gambia (R^2^ = 0.751, RMSE = 9.08 μg/m^3^, MAE = 4.39 μg/m^3^) – (Fig. 3a) – and Mozambique (R^2^ = 0.703, RMSE = 7.24 μg/m^3^, MAE = 3.79 μg/m^3^) – (Fig. 3c), indicating good agreement between observed and predicted concentrations. Kenya showed comparatively lower predictive strength (R^2^ = 0.537, RMSE = 6.03 μg/m^3^, MAE = 3.26 μg/m^3^) – (Fig. 3b), although interpolation accuracy remained acceptable for spatial surface estimation. Mean Error (ME) values were close to zero across all country–pollutant combinations, indicating minimal systematic bias in predictions. Overall, the cross-validation results demonstrate that the IDW interpolation captured spatial variability in pollutant concentrations with moderate-to-strong predictive agreement across the three study sites, supporting the use of the interpolated surfaces for subsequent spatial exposure analyses.

### 3.3. Spatial structure of daily PM_2.5_ in the Gambia, Kenya and Mozambique study sites

**Fig. 4.**
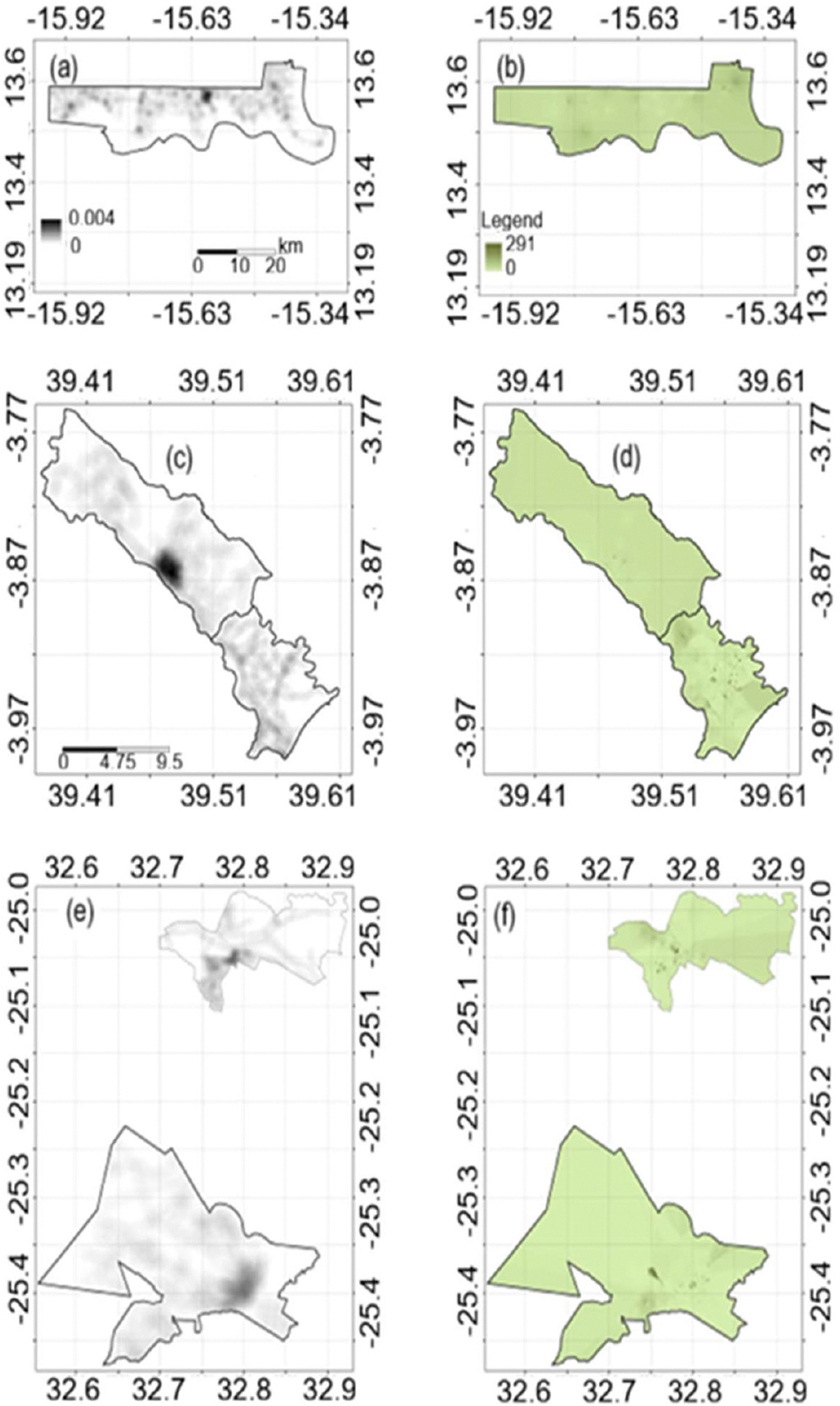
Spatial distribution of road density and mean daily PM_2·5_ exposure in the study sites. Panels (a–b) show road density and PM_2·5_ exposure in North Bank, The Gambia; panels (c–d) show Mariakani and Rabai, Kenya; and panels (e–f) show Manhiça District, Mozambique. PM_2·5_ concentrations represent interpolated mean daily exposure surfaces generated using IDW interpolation. Road density was derived from mapped road networks using geospatial density analysis in ArcGIS Pro. PM_2·5_ concentrations are expressed in μg m^−3^.

There was no clear association between road density and PM_2·5_ in The Gambia based on visual inspection of Fig. 4a and b. In Kenya, areas in the southwest with high road density in Fig. 4c do not show any association with PM_2·5_ (Fig. 4d). In Manhiça, high values of road network density were found in the eastern parts of the study area while low and almost uniform values were recorded elsewhere (Fig. 4e). Wider variations in PM_2·5_ (Fig. 4f) were observed in the eastern parts seemed associated with variations in the road network density which were more marked in the eastern parts than elsewhere.

### 3.4. Variations of PM_2.5_ concentrations between landcover types in study sites and comparison with WHO air quality guidelines

In The Gambia, mean daily PM_2·5_ concentrations were above 45 μg/m^3^ for all landcovers except bare and built-up areas (36–40 μg/m^3^), and overall levels were higher than in other sites (Table 2). In Kenya, PM_2·5_ values were higher in Rabai, reaching >41 μg/m^3^ in permanent water, grasslands, trees, and croplands, compared to >29 μg/m^3^ in Mariakani. In Mozambique, PM_2·5_ levels in Manhiça were the lowest (6–13 μg/m^3^), while Xinavane recorded higher values (>28 μg/m^3^) in built-up, bare, and water areas.

PM_2·5_ levels met interim Target 1 and Target 2 in all landcovers in all study sites in Gambia, Kenya and Mozambique. All landcovers in Mariakani in Kenya and in all study sites in Mozambique were meeting Target 3 for daily PM_2·5_ levels. However, in Gambia, Target 3 was only met in sparse vegetation areas while in Rabai, Kenya sparse vegetation, permanent and herbaceous wetlands had average daily PM_2·5_ greater than Target 3. Interim Target 4 for daily PM_2·5_ levels was met in all landcovers in Manhiça. In other study sites, daily PM_2·5_ levels were higher than Target 4 and exceeded the recommended daily AQG threshold (15 μg/m^3^). PM_2·5_ levels within recommended values in most landcovers in Manhiça except in built-up and herbaceous wetlands areas.

### 3.5. Road edge effect on PM_2.5_ concentrations for sites in Gambia, Kenya and Mozambique

**Fig. 5.**
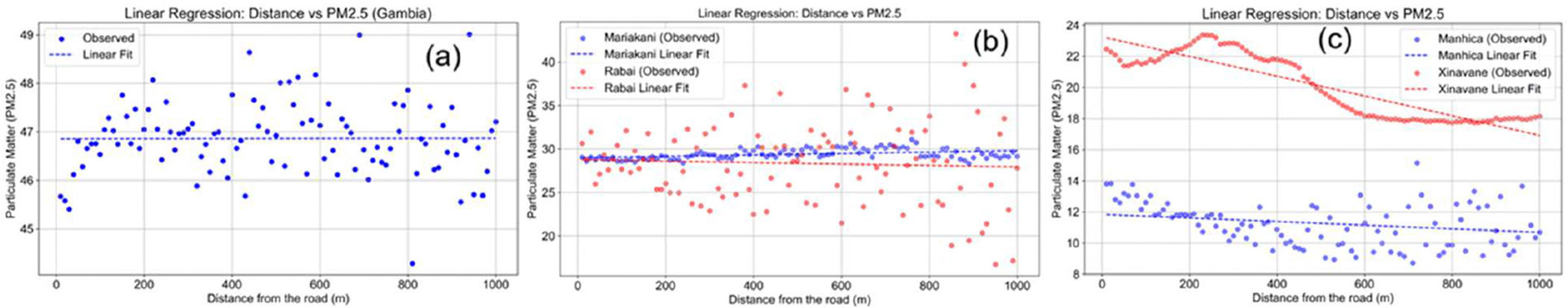
Road-edge effect on mean daily PM_2·5_ concentrations in The Gambia (a), Kenya (b), and Mozambique (c) based on distance-buffer analysis from major roads. PM_2·5_ concentrations were extracted from concentric road buffers generated at 10 m intervals up to 1000 m from road edges. Linear regression models were fitted to evaluate the relationship between PM_2·5_ concentration and distance from roads. Negative regression slopes indicate decreasing pollutant concentrations with increasing distance from roads, while positive slopes indicate increasing concentrations away from roads. R^2^ = coefficient of determination; CI = confidence interval; p = statistical significance level. PM_2·5_ concentrations are expressed in μg m^−3^.

In The Gambia, PM_2·5_ showed no significant linear relationship (β = 0.0013 μg m^−3^ per 100 m; 95% CI: −0.0503 to 0.0528; R^2^ ≈ 0; p = 0.962) – (Fig. 5a). In Mariakani (Kenya), PM_2·5_ increased modestly with distance (β = 0.0805 μg m^−3^ per 100 m; 95% CI: 0.0438 to 0.1172; R^2^ = 0.1585; p < 0.001), suggesting weaker explanatory power (Fig. 5b). In Rabai (Kenya), PM_2·5_ showed no statistically significant gradient (β = −0.0829 μg m^−3^ per 100 m; 95% CI: −0.4070 to 0.2411; R^2^ = 0.0026; p = 0.617). Xinavane (Mozambique) – (Fig. 5c), PM_2·5_ demonstrated a strong and highly significant decrease with distance (β = −0.6345 μg m^−3^ per 100 m; 95% CI: −0.6989 to −0.5701; R^2^ = 0.7919; r = −0.8899; p < 0.001), indicating a pronounced near-road effect. Manhiça (Mozambique), PM_2·5_ showed a weak but statistically significant decline (β = −0.1154 μg m^−3^ per 100 m; 95% CI: −0.2254 to −0.0054; R^2^ = 0.0414; p = 0.042). The magnitude and direction of the road edge effect varied considerably between locations, reflecting site-specific emission sources, atmospheric dispersion dynamics, and background pollution contributions.

## 4. Discussion of findings

The segmentation results highlight the importance of integrating movement characteristics with spatial contextual data when classifying exposure environments. Relying solely on speed-based thresholds can lead to substantial misclassification, particularly in settings where indoor movements may involve continuous activity such as walking within compounds, workplaces, or public facilities. However, it may be worth noting that the absolute GPS values may have contributed to exaggerated displacement in indoor environments potentially contributing to the misclassification. The incorporation of building footprint data significantly improves the reliability of indoor–outdoor classification and therefore strengthens the accuracy of personal exposure assessment. The differences observed across the study sites also suggest that exposure environments are strongly shaped by local settlement structure, housing density, and daily activity patterns. In locations where a larger proportion of time is spent indoors, exposure assessments that ignore indoor environments may underestimate important exposure pathways. Consequently, integrating spatial building datasets with mobility data is critical for improving environmental exposure modelling in diverse African urban and peri-urban contexts.

The interpolation analysis demonstrates the potential of spatial modelling approaches to generate reliable pollutant concentration surfaces in settings where monitoring networks are sparse. By producing spatially continuous estimates from limited observations, interpolation methods such as IDW can support detailed exposure assessments and enable the integration of environmental data with mobility-based exposure frameworks. The generally consistent model performance across the study sites indicates that spatial interpolation can effectively capture localized pollution gradients and support epidemiological and environmental health analyses. These findings highlight the value of combining spatial interpolation with high-resolution mobility and environmental sensing data to better understand spatial patterns of air pollution exposure, particularly in low- and middle-income countries where conventional monitoring infrastructure remains limited.

Highest daily average PM_2·5_ were recorded in permanent water, bare, herbaceous wetlands and built-up areas. Concentrations were generally low in deciduous open forests, deciduous broadleaf, closed and open forest areas. Mixed open forests had highest daily average PM_2·5_ concentrations while moderate values were observed in croplands. High values in built-up areas could include emissions from human activities such as household energy, fire smoke as well as vehicular emissions. In some circumstances, variations were observed for the same land covers in different study sites. For instance, in Mariakani, trees and grasslands had comparatively high PM_2·5_ values. Viippola et al. (2018) also observed higher PM_2·5_ concentrations in forests than open areas. Although vegetation enhances dry deposition of pollutants (Viippola et al., 2018), dense canopies may also reduce ventilation and inhibit horizontal dispersion, leading to localized accumulation of PM_2·5_ under low wind conditions (Vranckx et al., 2015). Consistent with our findings, Abhijith and Gokhale (2015) observed that different vegetation types and their configurations result in variations in interactions with pollutants and variations in concentration levels.

As the interim target threshold was reduced, the proportion of locations in the study sites not meeting these targets also increased. This implies that, generally, more time is still needed to formulate and implement emission reduction enough to meet interim targets as well as AQG. Additionally, daily concentrations of PM_2·5_ in the study areas still largely surpass WHO interim target and exceed the AQG level. Comparison with WHO daily air quality guideline thresholds (World Health Organization, 2021) suggests that pollutant levels in several study areas exceed concentrations associated with minimal health risk. These exceedances indicate potential public health concern and warrant further epidemiological investigation. However, this study did not perform quantitative health impact assessment or apply exposure-response functions; therefore, results should be interpreted as screening-level exposure characterization rather than definitive burden estimates. Although a few areas have reached AQG for one of the pollutants, fewer areas have reached the recommended minimum for both pollutants. This shows that the risk of adverse health outcomes associated with at least one of the pollutants is still high across much of the study sites in the three African countries. These findings underscore the need for expanded air quality monitoring and health-linked exposure assessment across data-scarce regions of sub-Saharan Africa. Efforts are still required to reach levels where PM_2·5_ cause minimal or no health challenges.

PM_2·5_ exhibited site-specific distance relationships. A strong and significant decline with distance was observed in Xinavane, whereas weaker or non-significant relationships were found in Rabai and The Gambia, suggesting limited dominance of traffic emissions in these areas. Although descriptive differences between roadside and distant buffers were observed in some locations (e.g., Rabai), the linear relationship was not statistically significant, indicating that distance alone does not explain PM_2·5_ variability at this site. Lawson et al. (2011) also observed elevated PM_2·5_ near road compared to far areas. This is in tandem with (Cardozo & Sánchez (2019)) which encountered increase in particulate matter towards road edges within a distance of 0.5 km from the road. (Cardozo & Sánchez (2019)) also affirmed that fine particulate has a gradual decay with distance from the road. Lin et al. (2016) also observed that people living 100 to 300m of main roads in Carolina USA had higher exposure to ultra fine particles than those away. High values of mean daily PM_2·5_ concentration were also found away from the road despite the general decrease with distance from the road. This may be attributable to PM_2·5_ releasing activities such as fires, agricultural activities and industries. Wind dispersion patterns may also transport super fine particles and concentrate them in low wind speed areas regardless of distance from the road. Although PM_2·5_ concentration generally decreased by 2 μg/m^3^ in 1000 m in Mariakani, the pattern was not uniform as values were elevated at some locations midway. This could indicate the possibility of other sources such as agricultural activities and emissions from the two sugarcane factories and dumpsites in the district. Presence of vegetation and other surfaces also alters variations of pollutants with distance from the road (Vranckx et al., 2015). Vranckx et al. (2015) alluded that tree canopies can form barriers between street level where the traffic pollutants are emitted and free flow aloft. In the Gambia study site, PM_2·5_ increased with distance in the first 500m from the road and decreased in the second 500m. These mid-distance variations were not captured by the linear model and likely reflect localized emission sources and dispersion dynamics. This could indicate the effect of either impediment of dispersion near roads such as by trees or polluting activities such as household energy and fire smoke pollution within settlements close to the roads or other polluting activities close to roads.

Monitoring periods differed across the three countries and did not encompass complete annual cycles. Seasonal influences such as biomass burning, agricultural activities, precipitation scavenging, and wind variability are known to significantly affect PM_2·5_ concentrations in sub-Saharan Africa. Consequently, the observed daily averages may not represent full-year exposure conditions. These findings should therefore be interpreted as representative of the monitoring windows rather than mean annual concentration. Future research incorporating year-round monitoring would strengthen long-term exposure characterization.

## 5. Limitations

Monitoring campaigns in The Gambia, Kenya, and Mozambique were conducted during specific periods rather than across a full annual cycle; therefore, the reported concentrations likely reflect conditions during the sampling windows and may not represent long-term annual exposure levels. Outdoor exposure was classified using a GPS-derived movement threshold (>1.5 m s^−1^), which may introduce some misclassification, particularly during slow outdoor activities or movement within large indoor environments. In addition, the interpolated exposure surfaces for PM2.5 depend on participant spatial coverage and interpolation assumptions, which may introduce uncertainty in areas with limited observations.

## 6. Conclusion

Based on the findings, it can be concluded that although mean daily PM_2·5_ concentrations meet interim targets in some areas of the study sites, the levels remain higher than the recommended minimum, posing significant health risks. The risk of PM_2·5_-related human health challenges therefore remain high for all study sites in Gambia, Kenya, and Mozambique. Results further show that pollutant levels interact in complex ways with land cover, with built-up, bare, water, and herbaceous wetland areas generally experiencing elevated concentrations. The variation of mean daily pollutant levels with distance from roads differed across the study sites, indicating that PM_2·5_ levels were not consistently higher near roads compared to farther away. This suggests that vehicular emissions, in combination with other sources, contribute to the overall exposure burden, and that distance from the road alone may not be a sufficient indicator of pollutant variation.

## 7. Recommendations

The study recommends further research to associate PM_2·5_ exposures with actual health outcomes in sub-Saharan Africa to evaluate the effectiveness of the WHO targets in reducing health challenges. It also suggests incorporating additional factors, such as emission reduction policies and strategies, when examining the relationship between pollution and distance from roads. Moreover, assessing edge effects for different types of roads, such as paved and unpaved, would provide a clearer understanding of spatial variations in pollutant levels. Finally, an in-depth analysis of the characteristics of vegetation in sub-Saharan Africa is necessary to explain the observed differences in PM_2·5_ concentrations across various vegetation types.

## Figures and Tables

**Table 1:** WHO daily PM_2·5_ concentration targets and air quality guideline thresholds used for health risk assessment.

Pollutant	Pollution reduction targets (μg/m^3^)				
	Target 1	Target 2	Target 3	Target 4	AQG
PM_2·5_	75	50	37.5	25	15

AQG = Air Quality Guideline recommended by the World Health Organization (WHO). PM_2·5_ concentrations are expressed in μg m^−3^. Interim targets (Target 1-4) represent progressive reduction thresholds intended to guide air quality improvement toward the AQG level.

**Table 2:** Mean daily PM_2⋅5_ concentrations across land use and land cover (LULC) classes in The Gambia, Kenya, and Mozambique and comparison with WHO guideline thresholds.

LULC	Mean daily PM_2⋅5_ concentration (μg m^−3^)				
	Gambia	Kenya (Mariakani)	Kenya (Rabai)	Mozambique (Manhiça)	Mozambique (Xinavane)
Tree cover	47.9	29.3	30.2	11.9	19.6
Shrubland	48.1	28.9	26.6	9.3	20.5
Grassland	45.1	29.6	26.3	12.1	19.9
Cropland	46.2	29.5	37.4	13.0	21.8
Built-up	39.6	27.8	30.8	15.9	29.6
Bare/sparse vegetation	36.6	29.1	41.5	7.7	29.4
Permanent water	45.2	31.3	73.0	6.9	28.9
Herbaceous wetland	48.4	-	48.0	15.8	17.5
Mangroves	46.5	-	-	-	-
Below AQG	Above AQG				

PM_2⋅5_ concentrations are expressed in μg m^−3^. AQG = World Health Organization Air Quality Guideline. Values exceeding AQG and interim targets indicate increased potential risk of adverse health outcomes. LULC = land use and land cover.

## Data Availability

The authors do not have permission to share data.
